# Analysis pipeline for the epistasis search – statistical versus biological filtering

**DOI:** 10.3389/fgene.2014.00106

**Published:** 2014-04-30

**Authors:** Xiangqing Sun, Qing Lu, Shubhabrata Mukherjee, Paul K. Crane, Robert Elston, Marylyn D. Ritchie

**Affiliations:** ^1^Department of Epidemiology and Biostatistics, Case Western Reserve UniversityCleveland, OH, USA; ^2^ Department of Epidemiology and Biostatistics, Michigan State UniversityEast Lansing, MI, USA; ^3^ Department of Medicine, University of WashingtonSeattle, WA, USA; ^4^ Department of Biochemistry and Molecular Biology, The Pennsylvania State University, University ParkPA, USA

**Keywords:** epistasis, genetic interaction, biological interaction, filtering pipeline, optimal search

## Abstract

Gene–gene interactions may contribute to the genetic variation underlying complex traits but have not always been taken fully into account. Statistical analyses that consider gene–gene interaction may increase the power of detecting associations, especially for low-marginal-effect markers, and may explain in part the “missing heritability.” Detecting pair-wise and higher-order interactions genome-wide requires enormous computational power. Filtering pipelines increase the computational speed by limiting the number of tests performed. We summarize existing filtering approaches to detect epistasis, after distinguishing the purposes that lead us to search for epistasis. Statistical filtering includes quality control on the basis of single marker statistics to avoid the analysis of bad and least informative data, and limits the search space for finding interactions. Biological filtering includes targeting specific pathways, integrating various databases based on known biological and metabolic pathways, gene function ontology and protein–protein interactions. It is increasingly possible to target single-nucleotide polymorphisms that have defined functions on gene expression, though not belonging to protein-coding genes. Filtering can improve the power of an interaction association study, but also increases the chance of missing important findings.

## INTRODUCTION

Genome-wide association studies (GWAS) and next generation sequencing association studies based on single marker tests can identify many associated genetic variants, but typically explain only a small portion of the total estimated heritability. Gene–gene interactions may play an important role in the genetic etiology underlying complex phenotypes and statistical analyses that consider interaction may increase the power to detect epistatic genetic associations, especially among low-marginal-effect markers.

Bateson (1909) defined epistasis as distortions from Mendelian segregation ratios due to one gene masking the effects of another. Fisher (1918) introduced the term “epistacy,” considering it to be any departure from a linear model in which the phenotypic effects of genotypes at two or more loci are assumed to be additive. Ever since, the terms “epistasis” and “gene–gene interaction” have often been used interchangeably and we make no distinction between these two terms here. However, the purpose of including such terms in any genetic model must be considered. If, for example, we know that segregation at each of two loci affects a particular phenotype, whether quantitative or binary, we already know there must be *biological* interaction. So, unless our purpose is to describe that interaction, no further analysis is necessary to *detect* its presence. In the case of a quantitative trait, whether or not there are interactions can depend on the scale of measurement, so the scale of the outcome is relevant. Factors that are additive with respective to the outcome measured on one scale may not be additive on another (Elston, 1961; Frankel and Schork, 1996; Greenland et al., 1998; Wang et al., 2010; Steen, 2012). Similarly, in the analysis of a binary trait, the link function used in a generalized linear model may determine whether or not interaction terms are necessary (Satagopan and Elston, 2012). If no transformation or change in link function can remove the interaction, it is called essential; in that case the best way to describe the interaction depends on how much of it is removable by a transformation or change of link function, and how much is essential. Simply describing the interaction by an appropriate statistical model may be useful for prediction in the same population as that sampled, but a prediction model may not be generalizable to other populations unless it is based on biological function.

Detecting pair-wise or higher-order statistical interactions can require enormous computational time. In a genome-wide analysis, the increased computational cost makes it impractical to examine whether interactions are non-essential or can be better described by removing non-additivity. Advances in computational methods, such as using a GPU framework (Yung et al., 2011; Zhu et al., 2013) and parallel computing strategies may overcome this limitation. However, the multiple hypothesis testing issue needs to be considered: this is the major reason why most existing epistasis studies are limited to searching for pair-wise interactions among a moderate number of genetic markers.

### STATISTICAL METHODS FOR DETECTING STATISTICAL INTERACTIONS

Regression-based approaches are mostly used to model and test interactions. The regression approach has been implemented in the epistasis module of PLINK (Purcell et al., 2007) to test pair-wise diallelic by diallelic epistasis for both quantitative and binary traits. An extension of the PLINK epistasis module, FastEpistasis, uses an efficient parallel computation algorithm to test pair-wise interactions. FastEpistasis is 15 times faster than PLINK using a single core computer (Schüpbach et al., 2010). Marchini et al. (2005) proposed an approach for joint association analyses allowing for pair-wise interactions based on logistic models; their approach uses an exhaustive search among single-nucleotide polymorphisms (SNPs) meeting some low marginal significance threshold. The software package PLATO can perform linear or logistic regression interaction analysis, calculating the full model, the reduced model, and the likelihood ratio test comparing the two (Grady et al., 2010).

The advantages of regression-based approaches are the clear interpretation of the model and the parameters that relate genotypes to phenotype. However, regression-based approaches have many technical and computational disadvantages for testing higher-order interactions and require many more tests: the number of parameters to be tested increases exponentially with the number of SNPs in the model.

Model-free approaches, such as machine learning and pattern recognition, afford an alternative strategy, and are capable of detecting high-dimensional non-linear interactions. This approach generally does not estimate parameters. It finds combinations of SNPs that can best separate cases and controls associated with the disease by epistatic interactions or joint effects. Some model-free approaches collapse high dimensional data into two dimensions, such as the combinatorial partitioning method (CPM; Nelson et al., 2001), restricted partition method (RPM; Culverhouse et al., 2004), set association (Wille et al., 2003), and multifactor dimensionality reduction (MDR;Hahn et al., 2003).

Unsupervised pattern recognition has also been used to detect interactions. Li et al. (2011) proposed a method for family based studies to detect differentially inherited SNP modules by hierarchically clustering SNPs that could be interactively associated with a disease. They first construct a genomic context-based SNP network based on adjacency on the chromosome. The association between each SNP and disease is evaluated on the basis of mutual information between SNP identity by descent sharing and affection status sharing of pairs of siblings. Then they use a hierarchical clustering algorithm to find risk SNP modules (clusters) for which discriminative scores are locally maximal. In each module, the SNPs are within a certain network distance (defined as the number of edges separating connected SNPs), and the discriminative score of a module is the maximum mutual information of the SNPs in the module, reflecting the risk associated with the module.

A likelihood ratio-based Mann–Whitney approach (Lu et al., 2012) and its extension (Wei et al., 2013) are other non-parametric methods for detecting interaction. They use a multi-locus Mann–Whitney statistic to evaluate the joint association of a SNP combination. Using a computationally efficient forward selection algorithm makes these methods feasible for genome-wide gene–gene interaction analyses. Nevertheless, they require at least one SNP in the combination to have a significant marginal association. The non-parametric approaches do not suffer from the issue of an increasing number of parameters when modeling high-order interactions, but it is difficult to determine how the detected SNP combinations affect the disease, either via the single marker associations or via their interactions.

Some studies test marker–marker interactions by testing linkage disequilibrium (LD) in the diseased population (Zhao et al., 2006), or test the contrast of LD or Pearson correlation in cases and controls (Kam-Thong et al., 2010; Prabhu and Pe’er, 2012). These methods are based on the idea that, if two unlinked markers are interactively associated with a disease, the two markers will have LD patterns in the disease population. If controls are not studied, these methods assume that the controls do not exhibit similar patterns.

### FILTERING PIPELINES FOR EPISTATIC INTERACTIONS PRIOR TO ANALYSIS

In GWAS, an exhaustive search among millions of SNPs for higher-order statistical interactions, or even just pair-wise interactions, could be computationally and statistically challenging. Filtering pipelines limit the number of tests performed between selected SNPs, whereas the use of computational technology and optimal algorithms increases the computational speed, and accelerates convergence if maximization is involved. While data driven filtering such as statistical filtering cleans the data to avoid the analysis of bad and least informative data, other types of filtering can be used purely to improve the power of interaction association analyses. In particular, filtering using biological knowledge limits the analysis to find the biologically most likely interactions.

#### Knowledge-driven filtering

Interaction models that are constructed based on specific biological knowledge are more likely to make sense. Research over the last several decades has accumulated vast amounts of biological information that is stored in public databases. These include gene ontology annotation, gene–gene interaction databases, pathways, disease related gene networks and systems, as shown in **Table 1**. This information can greatly assist GWAS to find epistatic interactions. Many recent studies have used such biological knowledge and databases for filtering in their interaction studies. The databases have helped identify biological pair-wise interactions among SNPs in pathways, and hence new associations and potential drug targets. For example, Liu et al. (2012) generated genome-wide SNP pairs based on multiple biological pathways such as KEGG, STRING, T2DGADB, etc.

**Table 1:** Biological information databases on gene ontology annotation, gene–gene interactions, pathways, disease related gene networks and systems.

Database	URL	Description	Reference
KEGG	http://www.genome.jp/kegg/pathway.html	KEGG is a collection of manually drawn pathway maps representing knowledge on the molecular interaction and reaction networks for metabolism, genetic information processing, environmental information processing, cellular processes, organismal systems, human diseases, and drug development.	Kanehisa and Goto (2000)
GO	http://www.geneontology.org/	GO provides an ontology of defined terms representing gene product properties. The ontology covers three domains: cellular component, molecular function, and biological processes.	Ashburner et al. (2000)
DIP	http://dip.doe-mbi.ucla.edu/dip/	Databases of experimentally determined interactions between proteins.	Xenarios et al. (2000)
BioGRID	http://thebiogrid.org/	A comprehensive resource of protein–protein and genetic interactions for all major model organism species.	Stark et al. (2006)
NetPath	http://www.netpath.org/	Resource of signal transduction pathways in humans.	Kandasamy et al. (2010)
IntAct	http://www.ebi.ac.uk/intact/	Database of molecular interactions that are derived from literature curation or direct user submissions.	Orchard et al. (2014)
MINT	http://mint.bio.uniroma2.it/mint/	MINT focuses on experimentally verified protein–protein interactions mined from the scientific literature by expert curators.	Chatr-aryamontri et al. (2007)
		MINT now uses the IntAct database infrastructure to limit the duplication of efforts and to optimize future software development.
MIPS	http://mips.helmholtz-muenchen.de/proj/yeast/CYGD/interaction/	The MIPS mammalian protein–protein interaction Database is a collection of manually curated high-quality interactions.	Pagel et al. (2005)
Pfam	http://pfam.sanger.ac.uk/	The Pfam database is a large collection of protein families, each represented by multiple sequence alignments and hidden Markov models. There are two kinds of entries in Pfam: Pfam-A entries are high quality, manually curated families; Pfam-B entries have lower quality.	Punta et al. (2012)
STRING	http://string-db.org	A database of known and predicted protein interactions, including direct (physical) and indirect (functional) associations.	Szklarczyk et al. (2011)
MSigDB	http://www.broadinstitute.org/gsea/msigdb/	Molecular signatures database, a collection of annotated gene sets integrating canonical pathways representing biological processes.	Subramanian et al. (2005)
BioCarta	http://www.biocarta.com/genes/	Includes classical pathways as well as current suggestions for new pathways.	Nishimura (2001)
Reactome	http://www.reactome.org/PathwayBrowser/	The Reactome pathway database aims to provide intuitive bioinformatics tools for visualization, interpretation and analysis of pathway knowledge.	Croft et al. (2011)
T2DGADB	http://t2db.khu.ac.kr:8080/	A disease gene network database for type 2 diabetes.	Lim et al. (2010)

Biofilter is an analysis pipeline that catalogs biological information by integrating data from the Reactome, KEGG, GO, DIP, Pfam, Ensembl, and NetPath (Bush et al., 2009; Pendergrass et al., 2013b). It can build SNP–SNP models based on known interactions between genes and proteins in curated pathways and networks. Grady et al. (2011) utilized the Biofilter software to look for epistasis contributing to the risk of virologic failure. Approximately two million SNP–SNP interaction models were produced by Biofilter, and Grady et al. (2010) tested these models by using logistic regression via the software package PLATO. They identified interactions between SNPs in the TAP1 and ABCC9 genes. Pendergrass et al. (2013a) identified five significant GxG interactions associated with cataract using Biofilter. Bush et al. (2011) studied multiple sclerosis susceptibility with Biofilter, identifying gene–gene interactions of susceptibility loci involved in the central nervous system and neuron function. Turner et al. (2011) used Biofilter to detect associations with low density lipoprotein cholesterol level, identifying 11 significant GxG interactions, eight of which were replicated in a second cohort. In each of these examples, Biofilter generated biologically plausible gene–gene and SNP–SNP interaction models that were replicated in an independent study.

Some studies reduce the number of tests by performing a gene-based, as opposed to a SNP-based, interaction test. Baranzini et al. (2009) combined the SNP-wise *P*-values to form a gene-wise *P*-value for each gene (such as using the minimum *P*-value for the gene), and superimposed the gene-wise *P*-values on a human protein interaction network to identify sub-networks containing a higher proportion of genes associated with multiple sclerosis than expected by chance. Ma et al. (2013) tested interactions of SNP pairs that are separately located in two different genes as marker-based tests. To test the interaction between each pair of genes, they combined these marker-based interactions and the LD between markers into a gene-based statistic.

Knowledge-driven filtering approaches can test models of genes that participate in the same biological pathway or network, and the interpretation of the interactions is then more straightforward. But their precision and power are hard to validate by simulation. Because such approaches depend on prior knowledge, which may not be accurate or may not be applicable to a particular dataset, they may miss what could be important findings among the genes for which we have little knowledge.

#### Data-driven filtering

Filtering based on statistical tests is data-driven. Statistical data-driven filtering includes, apart from SNP quality control, single marker associations, feature selection to keep only the most informative markers, and statistical tests to screen for potential interactions. Using data-driven filtering in GWAS can dramatically decrease the search space used to find interactions, so that subsequent statistical tests and machine learning methods can be applied as an exhaustive search among a smaller number of SNPs. The performance of data-driven filtering depends on the assumptions that the statistical tests or filtering algorithms make. Single marker association filtering can only screen interactions among SNPs showing at least a moderate effect on the trait of interest, while feature selection filtering and variance heterogeneity filtering can be used to detect SNP interactions with very weak marginal SNP effects.

***Filtering according to single marker association.*** Filtering SNPs based on their marginal effects is frequently used for a high-dimensional gene–gene interaction search. It is often combined with biological filtering to identify interactions among SNPs that are marginally associated with a phenotype (Baranzini et al., 2009; Grady et al., 2011; Turner et al., 2011; Ma et al., 2012; Pendergrass et al., 2013a). This approach follows the principles of hierarchical model building in the general linear model, where the interaction terms are tested only after all main-effect terms are deemed statistically significant. Typically the significance threshold used is less stringent than the usual genome-wide threshold of 5 × 10^-^^8^. The advantage of this filtering is that it is easy to implement; its disadvantage is that it has low power for detecting interactions among low-marginal-effect SNPs.

***Filtering by feature selection algorithms.*** Feature selection algorithms such as Relief (Kira and Rendell, 1992), ReliefF (Kononenko, 1994), Tuned ReliefF (TuRF; Moore and White, 2007), and Spatially Uniform ReliefF (SURF; Greene et al., 2009) can also be used. They screen pairs of diallelic SNPs that can cluster individuals with similar phenotypes, on the basis of the nine two-SNP genotypes, into two distinct classes (e.g., cases versus controls). For each individual only a small subset of neighboring individuals, i.e., individuals most similar to that individual over all the SNPs, is examined. Iterating over each individual and its chosen subset of neighboring individuals, SNPs are up-weighted for selection on the basis of belonging to the SNP pairs most frequently found in all such sets. Simulation results have indicated this is able to identify SNP pairs with purely non-additive effects in genome-wide datasets. Evaporative cooling (McKinney et al., 2007) is another feature selection approach which couples mutual information and thermodynamics theory. It filters SNPs by removing those with least information for epistatic interactions. Such feature selection filtering is able to retain pure epistatic (i.e., essential) interaction between markers with low-marginal effects, offering a powerful alternative to single-marker filtering.

***Filtering by testing variance heterogeneity of phenotype among SNP genotypes.*** For a quantitative trait, the presence of gene–gene interactions will result in heterogeneity of the phenotype variances among the genotypes of a single SNP, and this heterogeneity of phenotype variance has been proposed as a screen to prioritize SNPs for interaction testing (Paré et al., 2010; Struchalin et al., 2010). SNPs selected on the basis of variance heterogeneity would then be used for later gene–gene or gene–environment interaction analyses. However, unless the phenotypic means are the same for all the SNP genotypes, a transformation corresponding to a non-linear change in the scale of measurement may equalize the variances (Sun et al., 2013). This transformation, if it can be found, would eliminate any interactions detected this way.

### USING OPTIMAL SEARCH ALGORITHMS AND COMPUTATIONAL TECHNOLOGY TO SPEED A SCAN FOR INTERACTIONS

Exhaustive search of interactions among millions of SNPs in GWAS data is computationally time-consuming. However, heuristic stochastic searching algorithms and efficient computational technology, such as parallel computing and bit operation, can boost the computational speed and, if maximization is involved, speed the convergence required to calculate test statistics. Some interaction studies use optimal searching and computational technology to search the whole space for potential interactions. An ultrafast genome-wide scan approach for SNP–SNP interactions, SIXPAC, employs a randomization searching algorithm – probability approximate complete (PAC) testing – to drastically trim the universe of SNP combinations. The approach samples small groups of cases and highlights combinations of alleles carried by all individuals in the group. By further incorporating bit operation technology, SIXPAC can scan genome-wide pair-wise interactions in a few hours, compared to PLINK in weeks (Prabhu and Pe’er, 2012).

Lu et al. (2012) developed a likelihood ratio-based Mann–Whitney approach that can test high-order interactions. It is computationally efficient and only conducts one test for all the identified interaction, so that no adjustment is necessary for multiple testing. A further extension of the approach introduces a randomizing algorithm into the scan, using ensemble tree models (Wei et al., 2013), to increase the computational efficiency and convergence precision.

Schüpbach et al. (2010) developed an efficient extension of the PLINK epistasis module by using a parallel computing algorithm running on multiple processors to increase the speed of an exhaustive scan of all SNP pairs.

Heuristic or randomized search is much more efficient than exhaustive search, so it can perform a genome-wide scan of interactions among millions of SNPs without any filtering in reasonable time. However, it cannot guarantee reaching the optimal solution, which means it may not find all the biologically relevant interactions.

## CONCLUSION

Numerous approaches have been proposed for the analysis of epistatic interactions, each of which has advantages and disadvantages. Regression models are easy for model interpretation, but they are less suitable for modeling high-order interaction on a large number of markers. Model-free approaches do not give an explicit explanation of interaction findings, but they are good at detecting high dimensional non-linear interactions. Tests for interactions by contrasting LD between cases and controls or by studying phenotype variance heterogeneity among the different genotypes of a SNP, are two special tests for detecting epistasis in the absence of any main- effect.

With the emergence of massive amounts of genome sequencing data, developing efficient searching algorithms and filter pipelines are especially important. Heuristic searching is much faster than exhaustive searching, at the cost of missing some true positive results and finding more false positive results. Filtering pipelines based on biological knowledge have the advantage of providing a clearer biological explanation for the detected interactions, but the assumed knowledge may be limited and not error-free, in which case such filtering may also lead to testing some irrelevant interaction models and may miss novel and important signals. Data-driven filtering cleans the data by removing low quality and the least informative SNPs, but its performance depends on the underlying assumptions of the filter. Because statistical and biological filtering each has unique features, they should be viewed as complementary to, rather than as competing with, each other. Through novel approaches for filtering and modeling GxG interactions, we may identify more of the missing heritability for common, complex traits.

## Conflict of Interest Statement

The authors declare that the research was conducted in the absence of any commercial or financial relationships that could be construed as a potential conflict of interest.
