# Corrigendum: Analysis pipeline for the epistasis search – statistical versus biological filtering

**DOI:** 10.3389/fgene.2014.00350

**Published:** 2014-10-09

**Authors:** Shubhabrata Mukherjee

**Affiliations:** Department of Medicine, University of Washington, Seattle, WA, USA

**Keywords:** epistasis, genetic interaction, biological interaction, filtering pipeline, optimal search

My name was misspelled as Shubhabrata Mukheerjee. The correct spelling is Shubhabrata Mukherjee. The original article has been updated.

## Conflict of interest statement

The author declares that the research was conducted in the absence of any commercial or financial relationships that could be construed as a potential conflict of interest.

